# Effects of a Concurrent Mixed-Modality (Telerehabilitation and Face-to-Face) Exercise Rehabilitation Program in a Patient with Multiple Myeloma Prior to Spinal Cord Transplantation: A Case Study

**DOI:** 10.3390/curroncol32050282

**Published:** 2025-05-16

**Authors:** Juan Carlos Hernández-Sigüenza, Paula Blanco-Gimenez, Luis Baraja-Vegas, Josep López-Soler, Francisco Javier Falaguera-Vera, Eloy Jaenada-Carrilero, Juan Vicente-Mampel

**Affiliations:** Department of Physiotherapy, Medicine and Health Sciences School, Catholic University of Valencia, 46001 Torrent, Spain; juanca97j@mail.ucv.es (J.C.H.-S.); paula.blanco@ucv.es (P.B.-G.); luis.baraja@ucv.es (L.B.-V.); josep.lopez@ucv.es (J.L.-S.); eloy.jaenada@ucv.es (E.J.-C.); juan.vicente@ucv.es (J.V.-M.)

**Keywords:** multiple myeloma, exercise, prehabilitation, marrow autotransplant

## Abstract

**Introduction:** Multiple myeloma constitutes approximately 12% of hematologic malignancies and predominantly affects older adults, significantly compromising their quality of life. Although exercise interventions have shown benefits in oncology, evidence specific to MM remains limited and of low certainty. The presence of complex comorbidities in MM patients necessitates highly individualized approaches. Prehabilitation has emerged as a promising strategy to enhance functional capacity prior to autologous stem cell transplantation. This case study evaluates the feasibility of a personalized, scheduled exercise intervention delivered via telerehabilitation. **Intervention:** This case study seeks to examine the feasibility of implementing a personalized and scheduled exercise intervention within a telerehabilitation framework for a medically complex patient with multiple myeloma (MM). The 12-week prehabilitation protocol is designed to enhance physical function prior to autologous bone marrow transplantation by integrating therapeutic exercise targeting key parameters related to quality of life and clinical resilience, such as muscular strength, aerobic capacity, coordination, and overall well-being. The intervention includes concurrent training (strength and aerobic exercises) delivered 2–3 times per week, with aerobic activities conducted independently at home through a virtual format. Assessments were performed at baseline and post-intervention. **Results and conclusion:** A personalized exercise program, implemented through a hybrid model of in-person and telerehabilitation, is both feasible and safe. It has the potential to enhance physical function and quality of life in patients with multiple myeloma. Further research is necessary to validate these findings across broader patient populations.

## 1. Introduction

According to the ICD-10, multiple myeloma (MM) is classified under code C90.0. The reported incidence of MM ranges from 0.5% to 4.8% [1], accounting for approximately 12% of all hematologic malignancies [2]. The disease is most commonly diagnosed in individuals between 65 and 70 years of age, with rare cases occurring before 40 years of age [3]. Patients with MM experience the second lowest health-related quality of life among cancer patients, surpassed only by those with lung cancer [4]. Autologous transplantation is crucial for younger and fit patients with MM [5]. Exercise-based treatments have shown significant efficacy across various cancer types throughout the disease process, aiming to improve drug absorption [6], mitigate treatment-related side effects, and enhance patient independence and overall quality of life [7]. Engaging in physical activity has demonstrated benefits for cancer patients in general, and although there is some supporting evidence for patients with multiple myeloma [8], the findings remain inconclusive. Several reviews have highlighted a high risk of bias and low-certainty evidence, suggesting that current exercise programs may not consistently improve the quality of life in this specific population [9]. This evidence gap highlights the need for more rigorous studies and individualized approaches to treatment.

Patients with MM often face complex comorbidities, such as renal dysfunction, bone fragility, and cancer-related fatigue, which underscore the need for highly personalized and carefully tailored exercise protocols [10]. In response, strategies have been proposed to address these unique challenges, including greater community involvement, education in primary care, and improved accessibility to exercise and nutritional resources [11]. These approaches reinforce the growing recognition of supportive interventions that extend beyond the standard oncologic care. In this context, prehabilitation, which is defined as the process of enhancing a patient’s functional capacity before treatment, has emerged as a promising approach. Its primary objective is to prepare patients for surgery by enhancing resilience, ultimately improving postoperative outcomes [12]. Recent research supports the safety, feasibility, and clinical benefits of prehabilitation exercise in oncological populations, showing improved functional outcomes and reduced postoperative complications [13,14]. Additionally, integrating psychological support into these programs has strengthened emotional resilience, further increasing their effectiveness [12]. Although multiple studies have explored the safety and feasibility of exercise in patients with MM [6], robust evidence regarding its direct impact on health-related quality of life remains limited [9].

Therefore, more rigorous clinical trials are necessary to comprehensively evaluate the effects of individualized exercise interventions in this population [10]. Recent findings suggest that personalized exercise interventions, when tailored to the distinct physical and clinical profiles of patients with hematologic malignancies, can improve adherence and lead to sustained functional improvements [13]. This case study aims to investigate the feasibility of implementing a personalized and scheduled exercise intervention within a telerehabilitation framework for a medically complex patient with MM. Furthermore, it seeks to evaluate whether this approach can lead to measurable improvements in physical function—specifically in terms of strength, balance, and aerobic capacity—while minimizing the risk of adverse events commonly associated with physical activity in this patient population. We propose that this customized intervention will substantially enhance the functional physical capacities of patients prior to undergoing autologous stem cell transplantation. This improvement will be assessed using specific clinical outcomes, including measures of strength, balance, and aerobic capacity.

## 2. Materials and Method

### 2.1. Study Design

A single case report was designed for the present research based on the fundamental principles of the 2017 Declaration of Helsinki [15]. It was evaluated and approved by the Ethics Committee of the Catholic University of Valencia (ID: No.: UCV/2023-2024/119). Furthermore, the study was implemented based on the Care checklist [16].

### 2.2. Patient Information and Clinical Findings

The patient provided informed consent after reviewing the patient information sheet and agreeing to participate in the study. The intervention was implemented in a patient diagnosed with multiple myeloma and fractures of the T8, T11, T12 and L1 vertebrae, aged 50 years, with a BMI of 18.7. The patient exhibited pain and stiffness in the thoracolumbar area (VAS) and significant general weakness in terms of functionality and independence, with a PIPER scale of 8 and an SF-36 of 40.

### 2.3. Study Chronology

**Figure 1:**
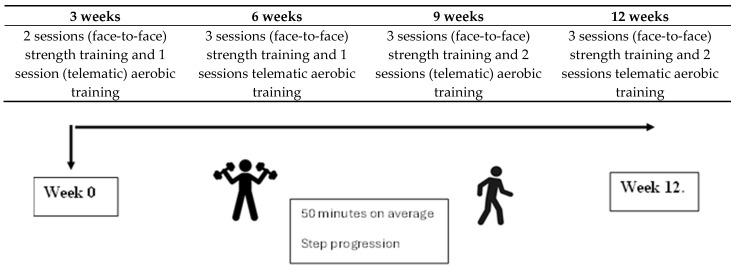
Study chronology.

This study was conducted from December 2023 to June 2024. The following evaluation and treatment phases were established (Figure 1). The therapeutic intervention was divided into 4 macrocycles of 3 weeks each. The duration of the sessions was 50 min on average.

### 2.4. Diagnostic Evaluation

The initial diagnostic evaluation was performed by an oncologist on 23 September 2024. Currently, the WHO in the IEC-10 classifies MM as a malignant hematological disease. The patient’s health status was assessed during the study through the FMS, 6MWT and STS (independence), Borg (fatigue), and SF-36 (quality of life) tests and various dynamometric evaluations (strength).

### 2.5. Therapeutic Intervention

The treatment combined in-person exercise with telerehabilitation sessions, as outlined below. A progression was implemented in terms of the number of weekly sessions and in terms of the loads and types of training work. The first session comprised an initial interview, education, data collection and first contact between patient and physiotherapist. Subsequently, the first three weeks consisted of 2 in-person training sessions per week and 1 online session. The following three weeks consisted of 3 in-person training sessions and 1 online session. Finally, the remaining 6 weeks comprised 3 in-person sessions and 2 online sessions. A table illustrating the proposal is presented below.

#### 2.5.1. Exercise Protocol

This section provides a detailed analysis of the entire exercise protocol, categorized by season and further subdivided into weekly segments. Each week’s activities and objectives are explicitly delineated to offer a structured approach to training. The protocol is designed to progressively enhance fitness levels, considering variations in intensity, volume, and recovery periods tailored to the specific demands of each season.

#### 2.5.2. Weeks 1–3

The following tables present the sessions for the initial three weeks. The online training comprised one day of ambulation in a level environment (the river of Valencia), progressing from 1500 steps to 3000 steps over the course of the three weeks (Table 1. Table 1 illustrates the parameters incorporated into the planning of each session.

#### 2.5.3. Weeks 4–12

From weeks 4 to 6, the program included one weekly session of ambulation on level terrain (the riverbed of Valencia), with a progressive increase from 3000 to 4000 steps. During the first week, the activity was divided into two sets with a mid-day rest, while by the final week, it was completed in a single continuous session. In weeks 7 to 9, the training frequency increased to two days per week, maintaining the same terrain. The participant performed between 4500 and 8000 steps per day, divided into two sessions with an interspersed mid-day rest period. Weeks 10 to 12 constituted a mesocycle focused on strength and speed development. The training continued with two weekly ambulation sessions, during which the participant completed between 8000 and 10,000 steps, again in two sets. The full schedule and session-by-session details are outlined in the Supplementary Material (Table S1).

### 2.6. Evaluation and Outcomes

The study variables are outlined below along with their respective measurement instruments. Age and clinical history were employed as discrete qualitative independent variables within a socio-demographic framework. Gender, derived from clinical history, was utilized as a nominal qualitative independent variable within the same socio-demographic context. Anthropometric data, including height, weight, BMI, muscle volumes, blood pressure, and oxygen saturation, were treated as continuous qualitative independent variables, measured using instruments such as a height rod, scale, measuring tape, blood pressure monitor, and oximeter.

#### 2.6.1. Dynamometer

The Camry (model EH101) hand-held dynamometer was utilized to measure grip strength. A decline in this strength is linked to restrictions in performing daily functional activities [17], with a reliability score of 0.88 [18].

#### 2.6.2. Sit-to-Stand Test

The sit-to-stand test is an effective tool for assessing individuals’ functional capacity, as the repeated action of rising from a chair is fundamental for daily tasks like walking, stair climbing, and standing. This test evaluates the ability to sit and stand (STS) from a chair over a period of 1 min [19,20] and has a reliability score of 0.80 [21].

#### 2.6.3. Berg Test

Balance was assessed using the Berg test, which is measured by the Berg balance scale. This tool is designed to evaluate balance by examining both dynamic and static aspects through 14 mobility-related tasks, each scored from 0 to 4. The scale is an effective predictor of fall risk, outcomes, and the duration of rehabilitation, and it can be administered quickly in various environments [22]. The test’s reliability is high, with inter-observer and intra-observer ICCs of 0.98 and 0.99, respectively [23].

#### 2.6.4. 6MWT

Resistance was assessed using the 6MWT [24], which evaluates the distance covered in six minutes on a 40 m track. Following the test, the participant indicated their perceived exertion on the 15-point Borg scale and reported leg muscle pain using the Category-Ratio (CR)-10 scale (0 = no pain, 10 = maximum pain) [25]. In individuals with cancer, the 6MWT is considered to be valid and reliable as it is for healthy older adults and those with cardiac and pulmonary conditions. Consequently, it is recommended for use in cancer patients [26].

#### 2.6.5. SF-36

The SF-36 is a prevalent and extensively utilized instrument for measuring health-related quality of life through 36 questions that are easy for patients to understand. These questions are designed to evaluate two primary aspects of a patient’s health: physical health and function and mental health and function. Within these categories, the questionnaire gauges the patient’s perceived health across eight broader items that include all 36 questions [27,28]. Answers are recorded using a Likert Scale, with scores ranging from 0 (indicating the worst perceived health) to 100 (indicating the best perceived health), where scores above or below 50 suggest better or poorer health, respectively [29,30,31]. The SF-36 scales demonstrated reliability exceeding the recommended standard (Cronbach’s alpha) of 0.7 in 96% of the assessments [28].

#### 2.6.6. PIPER

The Spanish adaptation of the PIPER [32] scale is a validated instrument consisting of 22 items designed to evaluate self-reported chronic fatigue among breast cancer survivors. This scale encompasses four aspects of subjective fatigue: “behavioral/severity”, “affective meaning”, “sensory”, and “cognitive/mood”. The scoring ranges from 0 to 10, with higher scores signifying increased fatigue levels. It has shown high reliability and validity, with a Cronbach’s alpha of 0.86 [33].

#### 2.6.7. FMS

The FMS is a series of tests designed primarily to evaluate a patient’s functional condition. Known as a functional movement screening test [34], this battery includes seven distinct assessments: deep squat, hurdle step, in-line lunge, shoulder mobility, straight-leg raise, trunk stability push-up, and rotary stability. These tests assess mobility, stability, and motor control [35]. Performance is scored from 0 to 3, taking into account any pain or compensatory movements, and the scores are totaled to a maximum composite score (CS) of 21 [36]. The FMS test has a sensitivity of 0.91 and a specificity of 0.54 [37].

### 2.7. Average Effort per Session

To tailor exercise intensity during treatment sessions on an individual basis, the Borg Rating of Perceived Exertion (RPE) was employed. The RPE is a widely utilized instrument for gauging an individual’s subjective perception of effort during physical activity. For patients with MM, the Borg Scale serves as a means to evaluate their perceived exertion levels during exercise or rehabilitation activities, thereby aiding in the prevention of excessive strain and in the standardization of exercise session load and volume. In the current study, the patient was instructed to maintain an RPE not exceeding 8 upon the completion of each exercise.

## 3. Results

The participant completed all prescribed sessions, with no adverse effects reported during the intervention. A total of 33 isolated exercise sessions were conducted throughout the period. Furthermore, 18 online sessions were independently organized to enhance the aerobic component.

### 3.1. Clinical Outcomes

#### 3.1.1. Descriptive Data

The results obtained after 12 weeks of implementing the exercise protocol are shown in Table 2.

#### 3.1.2. Percentage Change Analysis and Bar Charts

**Figure 2:**
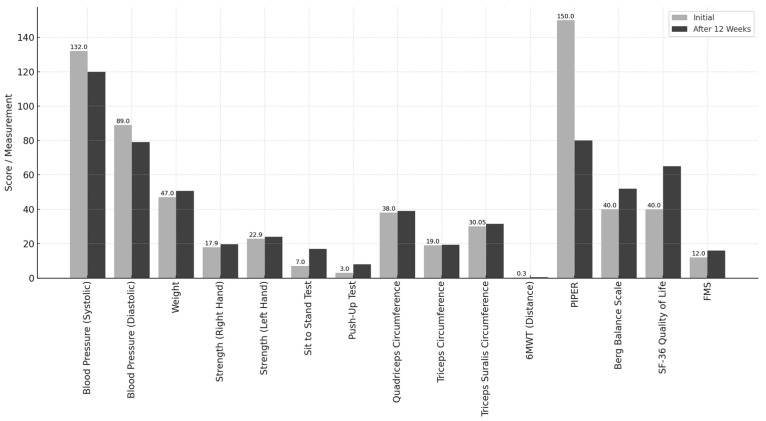
Pre- and post-intervention outcomes for key variables.

Blood pressure was reduced from 132/89 mmHg to 120/79 mmHg, representing a 9% decrease. Body weight increased from 47 kg to 50.6 kg, indicating a 7.6% gain. Muscular strength improved by 21.83% in the right hand and 22.44% in the left hand. The STST performance increased from 7 to 17 squats, reflecting a 58.82% improvement. The number of push-ups performed increased from 3 to 8 repetitions, indicating a 62.5% increase. The circumferences of the quadriceps, triceps, and calves increased by 1 cm, 1.5 cm, and 1 cm, respectively. The distance covered in 6MWT increased from 0.3 km to 0.45 km, reflecting a 50% improvement. The fatigue score on the PIPER scale decreased from 150 (68.18%) to 80 (36.36%). Balance, as measured by the Berg Balance Scale, improved by 21%. The SF-36 health survey score increased from 40% to 65%, indicating a 25% improvement. The FMS score increased from 12 (57.14%) to 16 (76.19%), representing a 19.05% improvement. We have also added pre–post evolution graphs (bar charts) that illustrate the initial and final values for each outcome. This visual representation allows for a clearer interpretation of the changes observed and is commonly used in single-subject designs to support descriptive findings (Figure 2).

### 3.2. Patient Perspective

From the patient’s perspective, several key aspects are noteworthy: “I initiated training during my chemotherapy cycles when my ability to walk was restricted to two blocks, and I was not participating in any physical activity. An initial evaluation indicated a considerable deficiency in strength and mobility. The initial sessions were brief; however, beginning in the third week, I observed significant improvements, which also contributed to an enhanced sense of general well-being. Consequently, we were able to extend the sessions to a full hour during the three-month preparation period for the first autotransplant. Gradually, equipment such as resistance bands was incorporated, and eventually, I was able to perform planks, squats, ascend and descend stairs, lift weights, and walk for extended periods as desired. In this context, we adhered to the training principles of progression and variability”. Another aspect of high clinical relevance was as follows: “This comprehensive approach significantly facilitated my ability to maintain a normal lifestyle, perform daily activities, and remain seated for the duration of a meal without the need for recumbency. It also expedited my recovery following the first autotransplant, as within three months post-procedure, and adhering to the same training regimen, I had achieved comparable levels of recovery and physical condition. In this context, it is imperative to consider the importance of rehabilitation, which enhances strength levels, thereby facilitating improved subsequent rehabilitation”.

## 4. Discussion

The preliminary clinical outcomes observed in this case study demonstrate improvements in key parameters, including muscular strength, fatigue, aerobic capacity, balance, and quality of life, following the implementation of a personalized and structured exercise program that integrated face-to-face sessions with a telerehabilitation component. These findings suggest that, even in a medically complex patient with MM, a hybrid intervention model may be both feasible and beneficial for enhancing physical function and overall well-being over a 12-week period. Recent evidence indicates that exercise positively impacts quality of life, functional independence, fatigue, and physical strength in patients with MM, thereby contributing to improved readiness for autologous bone marrow transplantation [38]. The clinical improvements observed in this case support the notion that individualized interventions enhance adherence and optimize outcomes. Over the course of 12 weeks, the patient exhibited substantial gains across multiple physical domains, aligning with prior research validating exercise as a safe and effective strategy for improving physical capacity in this population [6,7,39]. The feasibility of remotely delivering individualized exercise programs is particularly relevant in the context of MM, where patients frequently encounter challenges such as bone fragility, treatment-related fatigue, and immunosuppression.

A telerehabilitation model provides flexibility, limits exposure to clinical environments, and allows for consistent monitoring—key advantages for this vulnerable population. However, the effectiveness of such interventions depends on appropriate patient selection, comprehensive risk assessment, and sustained multidisciplinary collaboration involving physiotherapists, oncologists, and mental health professionals [40]. As illustrated in this case, the integration of gym-based and home-based exercises, under the supervision of a specialist cancer physiotherapist and supported by consistent follow-up, significantly contributed to the program’s success. Furthermore, resistance training tailored to individual capabilities was determined to be both feasible and safe for this patient. The implementation of a precision-based approach not only enhanced physical outcomes but also increased the patient’s self-efficacy in managing musculoskeletal discomfort, resulting in a reduction in analgesic use. Given the variability in symptom presentation and physical activity-related competencies in MM, exercise interventions should be meticulously adapted to individual needs. This includes appropriate levels of supervision and educational support tailored to the patient’s condition and preferences [41]. Consistent with the findings of this case, patients preparing for autologous stem cell transplantation often find physiotherapist-led evaluations and remotely supervised group exercise sessions to be acceptable. These strategies appear to enhance functional performance and quality of life.

A descriptive methodology, concentrating on percentage differences in pre- and post-intervention measurements to investigate changes in clinical outcomes were employed. Although this approach provides an initial perspective on the potential benefits and practical application of telerehabilitation-based interventions in MM, it possesses several limitations. The single-case design lacks inter-subject variability, thereby limiting the generalizability of the findings to the broader MM population. Furthermore, while visual outcome trends offer valuable insights into individual progress, they entail a degree of subjectivity and must be interpreted with caution. The absence of statistical inference further constrains the strength of the conclusions. Throughout the 12-week program, the patient demonstrated full adherence, attending all 33 scheduled supervised sessions and independently completing 18 additional telerehabilitation sessions. Notably, no adverse events were reported during the intervention, despite the concurrent administration of an intensive pharmacological regimen. These results highlight the potential safety and practicality of individualized exercise programs within telerehabilitation frameworks. Further research is necessary to evaluate the scalability, long-term safety, and clinical efficacy of such hybrid models in larger and more diverse populations of patients with multiple myeloma.

## 5. Conclusions

A personalized exercise regimen, implemented through a hybrid model that integrates in-person and telerehabilitation sessions, is feasible, safe, and potentially effective in enhancing the physical function and quality of life for a patient with multiple myeloma. These findings underscore the necessity for further research to validate the benefits of such interventions in larger and more diverse patient populations.

## Figures and Tables

**Table 1:** Exercise protocol session (weeks 1–3).

Season 1							
	Exercise	S	Rep	W	I	T	R
**Part 1**	*Air squats*	3	8		4/10	-	30″
	*Band pull apart* + *Y with theraband*	3	8 + 8		5/10	-	30″
	*Trunk rotation in sitting position*	3	10		3/10	-	30″
**Part 2**	*Balance*	3	3× (10 m forward and retun)		4/10	-	30″
	*Thoracic extensions*	3	8		5/10	-	30″
	*Front plank*	3	20 secs		7/10	-	30″
**Part 3**	*Education*	1					-

Exer (exercise); S (sets); Rep (repetitions); W (weight); I (intensity); T (time); R (rest).

**Table 2:** Summary of findings from the primary results at the start and conclusion of the follow-up period.

Outcome	Initial Value	After 12 Weeks	Difference (%)
Blood Pressure (Systolic/Diastolic)	132/89	120/79	9%
Weight	47 kg	50.6 kg	7.6%
Strength (Right Hand)	17.9 Kg/cm^2^	19.6 Kg/cm^2^	21.83%
Strength (Left Hand)	22.9 Kg/cm^2^	24 Kg/cm^2^	22.44%
STST (Sit to Stand Test)	7 squats	17 squats	58.82%
Push-Up Test	3 repetitions	8 repetitions	62.5%
Quadriceps (Circumference)	38 cm	39 cm	2.6%
Triceps (Circumference)	19 cm	19.30 cm	8.5%
Triceps suralis (Circumference)	30.05	31.5	2.6%
6MWT (Distance)	0.3 km	0.45 km	50%
PIPER	150 (68.18%)	80 (36.36%)	31.82%
Berg (Balance Scale)	40	52	21%
SF-36 (Quality of Life Scale)	40	65	25%
FMS	12 (57.14%)	16 (76.19%)	19.05%

## Data Availability

The data presented in this study will be available on request from the corresponding author.

## Supplementary Materials

The following supporting information can be downloaded at https://www.mdpi.com/article/10.3390/curroncol32050282/s1. Table S1: Illustration of the parameters incorporated into the planning of each session.
