# Supplementary material for: Effects of a Concurrent Mixed-Modality (Telerehabilitation and Face-to-Face) Exercise Rehabilitation Program in a Patient with Multiple Myeloma Prior to Spinal Cord Transplantation: A Case Study
Source: Curr Oncol. 2025 May 16;32(5):282. doi: 10.3390/curroncol32050282 (PMC12109700; doi:10.3390/curroncol32050282)
Supplement: Supplementary file 1 [file curroncol-32-00282-s001.zip › curroncol-3539933-Supplementary Materials V3.pdf]

Supplementary Material (Table S1)

Exercise protocol session. (Weeks 1-3).

Season 2							
	**Exercise**	**S**	**Rep**	**W**	**I**	**T**	**R**
**Part 1**	*Dead lift with elastic band*	3	8		4/10	-	30″
	Isometric squat 90º	3	30 secs		7/10	-	30″
	Bench press with elastic band	3	10		4/10	-	30″
**Part 2**	*Lunges*	3	8/side		8/10	-	30″
	Heel rise	3	15		6/10	-	30″
	Vertical pull with elastic band	3	10/side		5/10	-	30″

Exer (Exercise); S (Sets); Rep (Repetitions); W (Wheight); I (Intensity); T (Time); R (Rest)

Exercise protocol session (Weeks 4-6).

Season 1							
	**Exercise**	**S**	**Rep**	**W**	**I**	**T**	**R**
**Part 1**	*Dead lift with elastic band*	3	8		4/10	-	30″
	Isometric squat 90º	3	30 secs		7/10	-	30″
	Bench press with elastic band	3	10		4/10	-	30″
**Part 2**	*Lunges*	3	8/side		8/10	-	30″
	Heel rise	3	15		6/10	-	30″
	Vertical pull with elastic band	3	10/side		5/10	-	30″

Exer (Exercise); S (Sets); Rep (Repetitions); W (Wheight); I (Intensity); T (Time); R (Rest)

Exercise protocol session (Weeks 4-6).

Season 2							
	**Exercise**	**S**	**Rep**	**W**	**I**	**T**	**R**
**Part 1**	*Dead lift with elastic band*		15		4/10	-	30″
	Isometric squat 90º + lateral deltoid raise with dumbbell		60 secs		6/10	-	30″
	Push ups with knees		6		7/10	-	30″
**Part 2**	*Lunges*		8/side		8/10	-	30″
	Glute bridge 2 legs + 1 legs		10+10+ 10		6/10	-	30″
	Pull + rotation with elastic band		12/side		5/10	-	30″

Exer (Exercise); S (Sets); Rep (Repetitions); W (Wheight); I (Intensity); T (Time); R (Rest)

Exercise protocol session (Weeks 4-6).

Season 3							
	**Exercise**	**S**	**Rep**	**W**	**I**	**T**	**R**
**Part 1**	*Transversus abdominis in quadruped position*	3	8		6/10	-	30″
	Lateral plank	3	30 secs/side		5/10	-	30″
	Bench hamstring bridge	3	10		7/10	-	30″
**Part 2**	*Dynammic dead bug with dumbbell*	3	10/side		7/10	-	30″
	Up and down stairs	3	10+10+10		7/10	-	30″
	Monster walks + variant	3	20+12/side		5/10	-	30″

Exer (Exercise); S (Sets); Rep (Repetitions); W (Wheight); I (Intensity); T (Time); R (Rest)

Exercise protocol session (Weeks 7-9).

Season 1							
	**Exercise**	**S**	**Rep**	**W**	**I**	**T**	**R**
**Part 1**	*Squat 3-0-0*	3	12/side		8/10	-	30″
	Scapular retraction and protection in quadruped position	3	8		6/10	-	30″
	Stargered Squat + press militar con mancuernas 3-0-0	3	10	4kg	7/10	-	30″
**Part 2**	*Y balance test + dual task*	3	20		4/10	-	30″
	Bulgarian squat 3-0-0	3	8/leg		7/10	-	30″
	Floor press+ glute bridge	3	12		5/10	-	30″
**Part 3**	*Triceps extension with elastic*	3	12		5/10		
	Monster walks	3	20/side		5/10		
**Part 4**	*Education*	1					-

Exer (Exercise); S (Sets); Rep (Repetitions); W (Wheight); I (Intensity); T (Time); R (Rest)

Exercise protocol session (Weeks 7-9).

	Season 2						
	**Exercise**	**S**	**Rep**	**W**	**I**	**T**	**R**
**Part 1**	*Transversus abdominis in quadruped position*	3	8		4/10	-	30”
	Row with 2 hands	3	12		3/10	-	30”
	Bench hamstring bridge 1 leg 0-0-2	3	6		7/10	-	30”
**Part 2**	*Dead bug dynamic with dumbbell*	3	12/side		4/10	-	30”
	Vertical pull 1 hand	3	12/side		7/10	-	30”
	Glute Bridge 0-0-2	3	30 secs	10	6/10	-	30”
**Part 3**	*Dead lift with dumbbell*	3	12	6	6/10		
	Unilateral Farmer carry	3	3x (10 m forward and retun)	4	4/10		
	McKenzie extensions	3	8		3/10		
**Part 3**	*Education*	1					-

Exer (Exercise); S (Sets); Rep (Repetitions); W (Wheight); I (Intensity); T (Time); R (Rest)

Exercise protocol session (Weeks 7-9).

	Season 3						
	**Exercise**	**S**	**Rep**	**W**	**I**	**T**	**R**
**Part 1**	*Lateral Plank*	4	30 secs		5/10	-	30”
	Dynamic front plank	4	40 secs		3/10	-	30”
	Isometric Dead bug	4	10		7/10	-	30”
**Part 2**	*Paloff in isometric lunge*	3	3x (10 m forward and return)		4/10	-	30”
	Pull over	3	10	Disc 5	7/10	-	30”
	Static bike	3	3 minutes		8/10	-	30”
**Part 3**	*Education*	1					-

Exer (Exercise); S (Sets); Rep (Repetitions); W (Wheight); I (Intensity); T (Time); R (Rest)

Exercise protocol session (Weeks 10-12).

	Season 1						
	**Exercise**	**S**	**Rep**	**W**	**I**	**T**	**R**
**Part 1**	*Single leg squat (100°)*	4	8/side		8/10	-	30”
	Band pull apart + Y with elastic	4	8+8		3/10	-	30”
	Thruster	4	10	Dumbbells 4kg	7/10	-	30”
**Part 2**	*Balance gait with 90° hip flexion + anterior-posterior toe raises*	4	3x (10 m forward and return)		4/10	-	30”
	Squats with kettelbell	4	12		7/10	-	30”
	Glute bridge + triceps press with dumbbell	4	12	Dumbbells 3kg	7/10	-	30”
**Part 3**	*Push ups without knees + with knees*	4	2+5		9/10		-
	Hip abduction in monopodal support	4	15		6/10		
	Pull over with elastics	4	12		5/10		

Exer (Exercise); S (Sets); Rep (Repetitions); W (Wheight); I (Intensity); T (Time); R (Rest)

Exercise protocol session (Weeks 10-12).

	Season 2						
	**Exercise**	**S**	**Rep**	**W**	**I**	**T**	**R**
**Part 1**	*Hamstring curl with elastic*	4	12		7/10	-	30″
	inclined bench rowing with dumbbell	4	10		7/10	-	30″
	Biceps with dumbbell	4	10	4kg	5/10	-	30″
**Part 2**	*Unilateral Farmer carry*	4	3x (10 m forward and return)		4/10	-	30″
	Dinamic Paloff with elastic (power)	4	8/leg		5/10	-	30″
	Hip thrust 1 leg (power)	3	30 secs		8/10	-	30″
**Part 3**	*Dead lift with bar*	3	10	10	7/10		-
	McKenzie extensions	3	10		5/10		

Exer (Exercise); S (Sets); Rep (Repetitions); W (Wheight); I (Intensity); T (Time); R (Rest)

Exercise protocol session (Weeks 10-12).

	Season 3						
	**Exercise**	**S**	**Rep**	**W**	**I**	**T**	**R**
**Part 1**	Lateral Plank	4	30 secs		5/10	-	30”
	Dynamic front plank	4	40 secs		3/10	-	30”
	Isometric dead bug isométrico	4	10		7/10	-	30”
**Part 2**	Paloff in lunge	3	3x (10 m forward and return)		4/10	-	30”
	Pull over	3	10	Disc 5	7/10	-	30”
	Static bike	3	3 minutes		8/10	-	30”

Exer (Exercise); S (Sets); Rep (Repetitions); W (Wheight); I (Intensity); T (Time); R (Rest)
